# Comparative expression profile of microRNAs in *Anopheles anthropophagus* midgut after blood-feeding and *Plasmodium* infection

**DOI:** 10.1186/s13071-017-2027-6

**Published:** 2017-02-16

**Authors:** Wenquan Liu, Zhenhua Hao, Liyang Huang, Lingzi Chen, Qimei Wei, Liya Cai, Shaohui Liang

**Affiliations:** Department of Parasitology, Wenzhou Medical University, 325035 Wenzhou, Zhejiang Province People’s Republic of China

**Keywords:** MicroRNA (miRNA), *Anopheles anthropophagus*, Midgut, Blood-feeding, *Plasmodium* infection

## Abstract

**Background:**

*Anopheles anthropophagus* is one of the major vectors of malaria in Asia. MicroRNAs (miRNAs) play important roles in cell development and differentiation as well as in the cellular response to stress and infection. In a former study, we have investigated the global miRNA profiles in relation to sex in *An. anthropophagus.* However, the miRNAs contributing to the blood-feeding and infection with *Plasmodium* are still unknown.

**Methods:**

High-throughput sequencing was performed to identify miRNA profiles of *An. anthropophagus* midguts after blood-feeding and *Plasmodium* infection. The expression patterns of miRNA in different midgut libraries were compared based on transcripts per million reads (TPM), and further confirmed by Northern blots. Target prediction and pathway analysis were carried out to investigate the role of regulated miRNAs in blood-feeding and *Plasmodium* infection.

**Results:**

We identified 67 known and 21 novel miRNAs in all three libraries (sugar-feeding, blood-feeding and *Plasmodium* infection) in *An. anthropophagus* midguts. Comparing with the sugar-feeding, the experssion of nine (6 known and 3 novel) and ten (9 known and 1 novel) miRNAs were significantly upregulated and downregulated respectively after blood-feeding (*P* < 0.05, fold change ≥ 2 and TPM ≥ 10). *Plasmodium* infection induced the expression of thirteen (9 known and 4 novel) and eleven (9 known and 2 novel) miRNAs significantly upregulated and downregulated, respectively, compared with blood-feeding. The representative upregulated miR-92a in blood-feeding and downregulated miR-275 in *Plasmodium* infection were further confirmed by Northern Blot. Putative targets of these regulated miRNAs were further investigated and classified into their pathways.

**Conclusions:**

This study suggests that miRNAs are involved in the blood-feeding and *Plasmodium* infection in *An. anthropophagus* midgut. Further studies of the function of these differential expressed miRNAs will facilitate in better understanding of mosquito biology and anti-parasite immunity.

**Electronic supplementary material:**

The online version of this article (doi:10.1186/s13071-017-2027-6) contains supplementary material, which is available to authorized users.

## Background

Malaria, caused by *Plasmodium* parasites, is a major threat to human health worldwide. According to the reports from WHO, there were 214 million new infection cases of malaria, and about 438,000 malaria deaths worldwide in 2015 [1]. *Plasmodium* spp. are transmitted to humans by the blood-feeding of *Anopheles* mosquitoes. To accomplish the life-cycle, the *Plasmodium* must overcome numerous attacks from the innate immunity system in mosquitoes [2, 3]. Mosquitoes also have developed various mechanisms to confront *Plasmodium* infection [3, 4]. The midgut represents the first barrier for the survival and development of *Plasmodium* [4–6]. A key concept that has emerged from recent studies is the molecular mechanisms of mosquito midgut that negatively and positively modulate the invasion of the parasite [5, 6].

MicroRNAs (miRNAs) are 18–24 nucleotides non-coding RNAs that regulate gene expression at the post-transcriptional level [7]. They are now considered as a key mechanism of gene regulation in many cellular processes including development, differentiation, apoptosis and innate immunity [8–10]. miRNAs are also involved in the physiological functions of mosquitoes, such as sexual differences and blood-feeding, even in the control of viral and parasitic infections [11–16]. Until now, the miRNA profiles of midguts from *Anopheles gambiae* and *Anopheles stephensi* have been reported, and several miRNAs expression levels were shown to be altered during the response to blood-feeding and *Plasmodium* infection [13, 15–18]. For example, blood-feeding and *Plasmodium* infection in *An. stephensi* revealed regulation of 13 and 16 miRNAs respectively [18]. *Anopheles anthropophagus* is a species of mosquito that sucks human blood and transmits malaria (*Plasmodium vivax* and *Plasmodium falciparum*) as well as *Brugia malayi* filariasis in Southeast Asia [12, 19, 20]. The role of miRNAs in *An. anthropophagus* during blood-feeding and *Plasmodium* infection are still unknown.

In this study, we employed small RNA sequencing to identify miRNA expression profiles from three samples of *An. anthropophagus* midguts: sugar-feeding, blood-feeding and *Plasmodium* infection. The differential expression of miRNAs were further analyzed by target prediction and pathway analysis to reveal their roles in blood-feeding and *Plasmodium* infection. Our results provide novel regulated miRNAs information of *An. anthropophagus* during blood-feeding and parasite infection. Understanding the functions of these regulated miRNAs will help investigate mosquito biology and control mosquito-borne infectious diseases.

## Methods

### Mosquito rearing and *Plasmodium* infection

*Anopheles anthropophagus* (China wild type strain) was reared and maintained in humidified incubators at 26 ± 1 °C on a 12 h light:dark cycle as described previously [12]. Mice (18–20 g male ICR mice) were used for mosquito blood-feeding and *Plasmodium* infection experiments. Stock solution of *Plasmodium berghei* ANKA strain (0.2 ml) were thawed and intraperitoneally (i.p.) injected into ICR mice by using a 1.0 ml tuberculin syringe. When the gametocytes were confirmed, naive 4–5 day-old female mosquitoes were fed on *P. berghei-*infected or uninfected ICR mice. Mosquitoes were kept at 26 ± 1 °C until dissection.

### Mosquito dissections and total RNA isolation

Dissection of female adults midgut was performed 48 h post-blood-feeding on *Plasmodium*-infected or uninfected ICR mice. Adult female mosquitoes with 10% sugar-feeding were collected as control sample at 48 h. Dissections were performed on ice in RNAlater® Stabilization Solution (Ambion, Austin, U.S.A) and kept on ice. The midguts were dissected from the abdomen as described before [11]. Total RNAs were extracted from dissected tissue using mirVana^TM^miRNA Isolation Kit (Ambion). Quality and quantity of RNA was checked by using denaturalization agar gels and Du530 Spectrophotometer (Beckman, Krefeld, Gemany).

### Small RNA sequencing

The small RNA samples were collected and subsequently sequenced by illumina Hiseq2000 as described before [12]. Briefly, the small RNAs were ligated with RNA adapter followed by reverse transcription using RT primers. Following PCR amplification of the adaptor enriched fragments, the PCR-amplified cDNAs were size-selected using electroelution to obtain the small RNA population with length 119–134 bp. These small RNA libraries were then sequenced using illumina Hiseq2000 (BGI, China). There were 3 biological replicates for each library sequencing.

### Computational analysis of small RNA sequencing data

Raw reads generated by high-throughput sequencing was processed as previously described by us [11, 12] with slight modifications. First, low quality reads and reads smaller than 15 nucleotides (nt) were removed from the three small RNA read datasets of midguts, respectively. Clean reads derived from deep sequencing were trimmed and filtered with BOWTIE software to fetch sequences having an appropriate length (15–32 nt). Mature and pre-miRNA sequences of available mosquito species, i.e. *Anopheles gambiae* and *Aedes aegypti*, were used as a reference miRNAs database from miRBase v.21. Identification of the novel miRNA was performed by using RNA fold and miRDeep2 as described before [12]. The small RNA datas have been submitted to the NCBI/GEO database with the accession number GSE93545. Transcripts per million reads (TPM) for each miRNA in all three libraries was preformed to generate a comparative analysis of different midgut samples [17, 18]. A heatmap was generated by MeV software.

### Northern blot

Northern hybridizations were conducted using digoxigenin-labeled miRCURY LNA probes (Exiqon, Vedbak, Denmark) as described before [12]. Briefly, the midguts of female mosquitoes were collected at 48 h after blood-feeding. The total RNA sample was extracted using mirVana™ miRNA Isolation Kit (Ambion). Total RNA (20 μg) was loaded in 15% denaturing polyacrylamide gels. The RNA gels were transferred to a nylon membrane (Ambion), crosslinked using a UV crosslinker, and prehybridized, then hybridized overnight in the ULTRAhyb-Oligo Hybridization Buffer (Ambion) with the appropriate DIG-labeled probe at 42 °C. After washed three times, the membranes were then incubated for 5 min in development buffer. Substrate (1:100 diluted in development buffer) was applied on to the membranes and incubated in dark for 10 min. Chemiluminescence signal was then measured to detect miRNA on the membrane. Antisense 5′ digoxigenin-labeled miRCURY LNA probes sequences as follow, aan-miR-92a: 5′-TCA GCC GCT GTC ACA CGC ACA G-3′; aan-miR-275: 5′-GAC CAA TCG CCG TCC CCG CCG-3′.

### miRNA target prediction and pathway analysis

mRNA targets of regulated miRNAs during blood-feeding and *Plasmodium* infection were predicted according to the protocol reported before [18, 19]. Briefly, 3′UTR sequence of *An. gambiae* and *Ae. aegypti* were downloaded from VectorBase and subjected to target prediction using RNA hybrid tool. Target predictions were carried out based on the following three parameters: (i) the perfect complementarity of the miRNA with the 3′UTR sequence of mRNA targets; (ii) the energy of the miRNA:mRNA target duplex ≤ -20 Kcal/mol; (iii) *P*-value < 0.05. The selected miRNA:mRNA interaction networks were generated and visualized by Cytoscape.

### Statistical analysis

Statistical tests for identifying significant differentially expressed miRNAs were performed using *t*-test. The *P*-value cut-off was carried out on the data with the significance selected as 0.05.

## Results

### Small RNA sequencing analysis

**Fig. 1:**
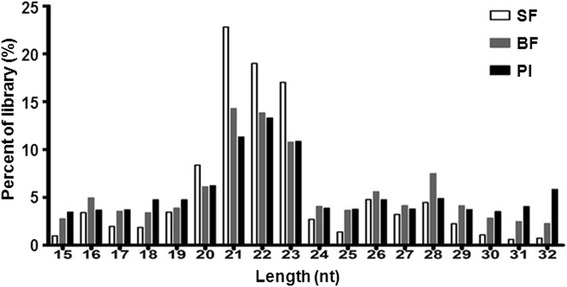
Length distribution of small RNA reads in the *An. anthropophagus* midguts. Female mosquitoes midguts at 48 h sugar feeding (SF), blood-feeding (BF) and *Plasmodium* infected-blood-feeding (PI)

The small RNA libraries were enriched, and separately yielded 17.51 million, 15.87 million and 6.5 million raw reads from the midgut samples of sugar-feeding (SF), blood-feeding (BF) and *Plasmodium* infection (PI). After filtering for linker sequences, and removing ambiguous reads, high quality clean reads with sizes ranged from 15 to 32 nt were collected, of which 14.45 million (82.56%) reads for SF, 11.75 million (74.02%) reads for BF, and about 3.2 million (50.38%) reads for PI (Table 1). The main populations of small RNA in the length distribution is 20–23 nt (Fig. 1), of which 10.1 million (68.89%) reads for SF, 5.76 million for BF (49.01%) and 1.45 million for PI (45.51%). The 20–23 nt peaks of small RNA are consisted with the expected size of microRNAs. After aligned to the known miRNA and pre-miRNA in miRBase (version 21.0), the miRNA reads from SF, BF and PI midguts which can match to the miRBase are 4.03 million, 1.88 million and 0.21million separately (Table 1).

**Table 1:** Composition of small RNAs in midguts of sugar-feeding (SF), blood-feeding (BF) and *Plasmodium* infected blood-feeding (PI) at 48 h

Sample name	Raw reads	Clean reads (%)	20–23 nt reads (%)	Reads matched to known miRNAs
SF	17,510,882	14,457,010 (82.56)	10,104,472 (68.89)	4,032,195
BF	15,875,249	11,750,726 (74.02)	5,759,187 (49.01)	1,878,567
PI	6,511,125	3,180,161 (50.38)	1,447,177 (45.51)	209,907
Total	39,897,256	29,387,897	17,310,836	6,120,669

### Identification of miRNAs in midguts of *An. anthropophagus*

A total of 67 known and 21 novel miRNAs were identified in the SF, BF and PI midgut libraries (Tables 2 and 3). Both known and novel miRNAs were present in the miRNA databases of *An. anthropophagus* adult as described before [12]. The distribution and relative abundance of these miRNAs were analyzed according to the set of “abundant” (TPM ≥ 1000) or “rare” (TPM < 10). Most of known and novel miRNAs were found in the SF library (*n* = 87) with 29 abundant and 20 rare miRNAs. In case of BF library, 26 miRNAs were found to be abundant and 7 miRNAs (miR-193, miR-2944b, miR-307, miR-309, miR-79-5p, miR-965-1 and miR-N6) were not detected. Meanwhile, 27 abundant and 20 rare miRNAs were found in the PI library, of which 7 miRNAs (miR-193, miR-210, miR-252, miR-263b-5p, miR-2944b, miR-307 and miR-87) have no read counts. A novel miRNA miR-N6 is unique in the PI midguts.

**Table 2:** List of known microRNAs expressed in *An. anthropophagus* midguts

				Transcripts per million reads(TPM)			Fold change		Reference miRNA database	
No.	Name	Length	Sequence	SF	BF	PI	BF *vs* SF	PI *vs* BF	aga	aae
1	aan-bantam	23	UGAGAUCACUUUGAAAGCUGAUU	45,048.5	24,928	74,181	0.553	2.975	Y	Y
2	aan-let-7	21	UGAGGUAGUUGGUUGUAUAGU	1,079.9	646.7	1,199.2	0.598	1.854	Y	Y
3	aan-miR-1*^#^	22	UGGAAUGUAAAGAAGUAUGGAG	78	20.6	75.5	0.264	3.665	Y	Y
4	aan-miR-10	22	ACCCUGUAGAUCCGAAUUUGUU	60.7	51.9	136	0.855	2.62	Y	Y
5	aan-miR-100^#^	22	AACCCGUAGAUCCGAACUUGUG	6,270.2	4632	17,444	0.738	3.766	Y	Y
6	aan-miR-1000	21	AUAUUGUCCUGUCACAGCAGU	56.6	129.1	115	2.280	0.89	Y	Y
7	aan-miR-11	22	CAUCACAGUCUGAGUUCUUGCU	3,794.7	1775.7	2,952.7	0.467	1.662	Y	Y
8	aan-miR-1174	21	UCAGAUCUACUUAAUACCCAU	4,040	1,674	3,367.6	0.414	2.011	Y	Y
9	aan-miR-1175-3p*	24	UGAGAUUCUACUUCUCCGACUUAA	7,421.2	2,027	4,887.2	0.273	2.411	Y	Y
10	aan-miR-12*^#^	23	UGAGUAUUACAUCAGGUACUGGU	5,206.8	1,768.2	13,763.1	0.339	7.783	Y	Y
11	aan-miR-125	22	UCCCUGAGACCCUAACUUGUGA	308.6	257.7	791.3	0.835	3.07	Y	Y
12	aan-miR-13*^#^	23	UAUCACAGCCAUUUUGACGAGUU	790.5	241.7	1,988.8	0.305	8.228		Y
13	aan-miR-133^#^	22	UUGGUCCCCUUCAACCAGCUGU	96.3	87.3	17.4	0.906	0.199	Y	Y
14	aan-miR-137	22	UAUUGCUUGAGAAUACACGUAG	20.1	14.8	34.9	0.736	2.358		Y
15	aan-miR-14^#^	22	UCAGUCUUUUUCUCUCUCCUAU	8,439	3,009.3	1,009.7	0.356	0.335	Y	Y
16	aan-miR-184	22	UGGACGGAGAACUGAUAAGGGC	6,949.9	4,432	9,130	0.637	2.06	Y	Y
17	aan-miR-1890	22	UGAAAUCUUUGAUUAGGUCUGG	112.6	41.8	80.2	0.371	1.918	Y	Y
18	aan-miR-1891	22	UGAGGAGUUAAUUUGCGUGUUU	3.8	3.2	3.5	0.842	1.093	Y	Y
19	aan-miR-190	24	AGAUAUGUUUGAUAUUCUUGGUUG	47	37.6	34.9	0.8	0.928	Y	Y
20	aan-miR-193	22	UACUGGCCUACUAAGUCCCAAC	0.2	0	0	0	–	Y	Y
21	aan-miR-210^#^	19	CUUGUGCGUGUGACAACGG	5.1	11.1	0	2.176	–	Y	Y
22	aan-miR-252*^#^	21	UAAGUACUAGUGCCGCAGGAG	59	192	0	3.254	–		Y
23	aan-miR-263a^#^	23	AAUGGCACUGGAAGAAUUCACGG	36.3	50.8	262.7	1.399	5.17	Y	Y
24	aan-miR-263b-5p	23	CUUGGCACUGGGAGAAUUCACAG	0.2	1.1	0	5.5	–		Y
25	aan-miR-275^#^	20	UCAGGUACCUGAAGUAGCGC	3,269.4	9,040.2	2,035.9	2.765	0.225	Y	Y
26	aan-miR-276*^#^	20	AGCGAGGUAUAGAGUUCCUA	23.9	7.4	27.9	0.309	3.770	Y	Y
27	aan-miR-277-5p	22	UAAAUGCACUAUCUGGUACGAC	15.3	7.7	14.9	0.503	1.935	Y	Y
28	aan-miR-278	23	ACGGACGAUAGUCUUCAGCGGCC	55.7	22.8	31.4	0.409	1.377	Y	Y
29	aan-miR-279*	22	UGACUAGAUCCACACUCAUUAA	2,787.5	926.2	1,746.5	0.332	1.885	Y	Y
30	aan-miR-283*	24	CAAUAUCAGCUGGUAAUUCUGGGC	1,231.3	407.5	693.7	0.330	1.702	Y	Y
31	aan-miR-285	22	UAGCACCAUUCGAAAUCAGUAC	10.4	23	18.6	2.211	0.808		Y
32	aan-miR-2944a	24	GAAGGAACUUCUGCUGUGAUCUGA	9.9	7.1	14.9	0.717	2.098		Y
33	aan-miR-2944b	23	GAAGGAACUCCCGGUGUGAUAUA	1.2	0	0	0	–		Y
34	aan-miR-2a^#^	23	UAUCACAGCCAGCUUUGAAGAGC	3,687.6	1,745.2	415.5	0.473	0.238		Y
35	aan-miR-2b*	24	UAUCACAGCCAGCUUUGAUGAGCU	791.7	269.1	618.2	0.339	2.297		Y
36	aan-miR-2c	23	UAUCACAGCCAGCUUUGAUGAGC	657.4	228	435.7	0.346	1.910		Y
37	aan-miR-305-5p	24	AUUGUACUUCAUCAGGUGCUCUGG	2,879.6	1,101.7	3,594	0.382	3.262	Y	Y
38	aan-miR-306	22	UCAGGUACUGGAUGACUCUCAG	14,095.1	11,671.1	11,518.1	0.828	0.986	Y	
39	aan-miR-307	20	UCACAACCUCCUUGAGUGAG	1.3	0	0	0	–	Y	Y
40	aan-miR-308-5p^#^	22	AAUCACAGGAGUAUACUGUGAG	85.4	39.7	338.2	0.464	8.518	Y	Y
41	aan-miR-309	22	UCACUGGGCAAAGUUUGUCGCA	0.2	0	3.5	0	–	Y	
42	aan-miR-31^#^	23	UGGCAAGAUGUUGGCAUAGCUGA	27.3	10.7	207	0.392	19.35		Y
43	aan-miR-315-5p*	22	UUUUGAUUGUUGCUCAGAAAGC	2.9	12.2	7	4.206	0.573	Y	Y
44	aan-miR-317	24	UGAACACAUCUGGUGGUAUCUCAG	6,342	6,462.2	2,795.8	1.018	0.432	Y	
45	aan-miR-34	20	UGGCAGUGUGGUUAGCUGGU	584.9	347.7	990.1	0.594	2.847	Y	Y
46	aan-miR-375-1	22	UUUGUUCGUUUGGCUCGAGUUA	10	6.9	20.9	0.69	3.028	Y	Y
47	aan-miR-71-3p	22	UCUCACUACCUUGUCUUUCAUG	279.6	174.3	100.6	0.623	0.577		Y
48	aan-miR-79-5p	22	UAAAGCUAGAUUACCAAAGCAU	1.4	0	3.7	0	–	Y	Y
49	aan-miR-8-5p	23	UAAUACUGUCAGGUAAAGAUGUC	1,990.3	3,340.8	2,245.8	1.678	0.672	Y	Y
50	aan-miR-87	21	GGUGAGCAAAUAUUCAGGUGU	3.3	2.6	0	0.787	0	Y	
51	aan-miR-927	22	UUUAGAAUUCCUACGCUUUACC	142	156.1	156.9	1.099	1.005	Y	
52	aan-miR-92a*	22	UAUUGCACUUGUCCCGGCCUAU	2,103.2	17,462.4	8,613.8	8.3	0.493	Y	
53	aan-miR-92b*^#^	22	AAUUGCACUUGUCCCGGCCUGC	503.4	2,215.5	366	4.40	0.165	Y	Y
54	aan-miR-932-3p*	23	UCAAUUCCGUAGUGCAUUGCAGU	1.7	10.4	7.4	6.117	0.711		Y
55	aan-miR-957^#^	22	UGAAACCGUCCAAAACUGAGGC	98.6	260.4	31.4	2.640	0.120	Y	Y
56	aan-miR-965-1	22	UAAGCGUAUAGCUUUUCCCAUU	2	0	7.4	0	–	Y	Y
57	aan-miR-970	21	UCAUAAGACACACGCGGCUAU	4,435.5	2,880.7	1,837.2	0.649	0.637	Y	Y
58	aan-miR-980^#^	24	CGGCCGUUCAUUGGGUCAUCUAGC	25.5	26.9	4.9	1.054	0.182		Y
59	aan-miR-981	22	UUCGUUGUCGACGAAACCUGCA	3.5	7.7	7.4	2.2	0.961	Y	Y
60	aan-miR-988-5p	22	CCCCUUGUUGCAAACCUCACGC	4	2.2	7.4	0.55	3.363	Y	Y
61	aan-miR-989*	21	UGUGAUGUGACGUAGUGGUAC	78	360	314	4.615	0.872	Y	Y
62	aan-miR-996*	21	UGACUAGAUUACAUGCUCGUC	6,082.2	1,855	5,933.3	0.305	3.198	Y	Y
63	aan-miR-998	19	UAGCACCAUGAGAUUCAGC	3.5	0.5	3.7	0.142	7.4		Y
64	aan-miR-9a	23	UCUUUGGUUAUCUAGCUGUAUGA	1,308.7	650.5	2,178.8	0.497	3.349	Y	Y
65	aan-miR-9b	24	UCUUUGGUGAUUUUAGCUGUAUGC	2,386.6	1,190.8	3,806.8	0.499	3.196	Y	Y
66	aan-miR-9c	22	UCUUUGGUAUUCUAGCUGUAGA	8,976.6	4,496.3	12,856.5	0.500	2.859	Y	Y
67	aan-miR-iab-4^#^	22	ACGUAUACUGAAUGUAUCCUGA	14.6	3.3	26.1	0.226	7.909	Y	Y

*Abbreviations*: *SF* sugar-feeding, *BF* blood-feeding, *PI Plasmodium* infection, *TPM* transcripts per million reads; Fold change, BF/SF or PI/BF, *aan Anopheles anthropophagus*, aga *Anopheles gambiae*, *aae Aedes aegypti*

*Represents the statistical significance in the differential expression of individual miRNA in BF *vs* SF (*P*-value < 0.05)

^#^Represents the statistical significance in the differential expression of individual miRNA in PI *vs* BF (*P*-value < 0.05)

**Table 3:** List of novel microRNAs expressed in *An. anthropophagus* midguts

				Transcripts per million reads(TPM)			Fold change	
No.	Name	Length	Sequence	SF	BF	PI	BF *vs* SF	PI *vs* BF
1	aan-miR-N1^#^	18	CGCUGCAGUACUGGCGCC	37.2	17.5	306.8	0.470	17.53
2	aan-miR-N2	21	AUCCGGUGAUAGGCUGACCCG	50.6	26.5	45.3	0.523	1.709
3	aan-miR-N3*	20	UUAGAAUGUGGAAUCUGUUU	27.9	7.4	24.4	0.265	3.297
4	aan-miR-N4	23	UUGGUGUUAUAUCUUACAGUGAG	3,160.5	2,462.1	3,824.2	0.779	1.553
5	aan-miR-N5	23	UUGGUGUUAUAUCUUACAGUGAG	9.8	4.2	10.5	0.428	2.5
6	aan-miR-N6^#^	20	UAUCACAGCCAGCUUUGAAG	0	0	149.9	–	–
7	aan-miR-N7	22	UGCAUUCAGUGGGGCGGUCGUG	4.2	4.8	3.5	1.142	0.729
8	aan-miR-N8	22	UGUUAACUGUAAGACUGUGUCG	5.1	1.1	7	0.215	6.363
9	aan-miR-N9	21	UAGCACCAUGAGAUUCAGCUC	794,875	861,632.1	704,445.5	1.083	0.817
10	aan-miR-N10*	22	UCAAUUCCGUAGUGCAUUGCAGU	80.5	273.1	491.5	3.392	1.799
11	aan-miR-N11	22	UUGGUGUUAUAUCUUACAGUGAG	1,479.9	592.8	348.6	0.400	0.588
12	aan-miR-N12	22	GUAGGCCGGCGGAAACUACUUGC	351.2	132.3	142.9	0.376	1.080
13	aan-miR-N13	23	UUGGCCGGUACGGGCUGACCGGGC	49.5	29.6	55.8	0.597	1.885
14	aan-miR-N14^#^	22	UGAACCGGCGUAGCGUGAAAGCA	8,461.5	3,769.9	18,898.1	0.445	5.012
15	aan-miR-N15	21	CUAAGUACUAGUGCCGCAGGAG	3,368.1	2,159.9	6,177.4	0.641	2.860
16	aan-miR-N16*^#^	19	UUAGAAUGUGGAAUCUGUUU	755.7	4,010.7	324.2	5.307	0.080
17	aan-miR-N17	22	UGAAAUCUUUGAUUAGGUCUGG	59.3	60.3	125.5	1.016	2.081
18	aan-miR-N18^#^	18	UAUCAGCGGUAGUUACCUG	22.7	13.8	3.5	0.607	0.253
19	aan-miR-N19	20	GUGCAUUGUAGUUGCAUUGCA	3,771.7	1,716.9	3,099.1	0.455	1.805
20	aan-miR-N20^#^	18	GUUGCUGUCCGCUGAAGCA	59.8	25.4	209.2	0.424	8.236
21	aan-miR-N21*	23	UGGCAAGAUGUUGGCAUAGCAGCU	14.2	97.6	134.1	6.873	1.373

*Abbreviations*: *N* the abbreviation of Novel, *SF* sugar feeding, *BF* blood feeding, *PI Plasmodium* infection, *TPM* transcripts per million reads; Fold change, BF/SF or PI/ BF, *aan Anopheles anthropophagus*

*Represents the statistical significance in the differential expression of individual miRNA in BF *vs* SF (*P*-value < 0.05)

#Represents the statistical significance in the differential expression of individual miRNA in PI *vs* BF (*P*-value < 0.05)

### Differential expression of miRNAs after blood-feeding and *Plasmodium* infection

**Fig. 2:**
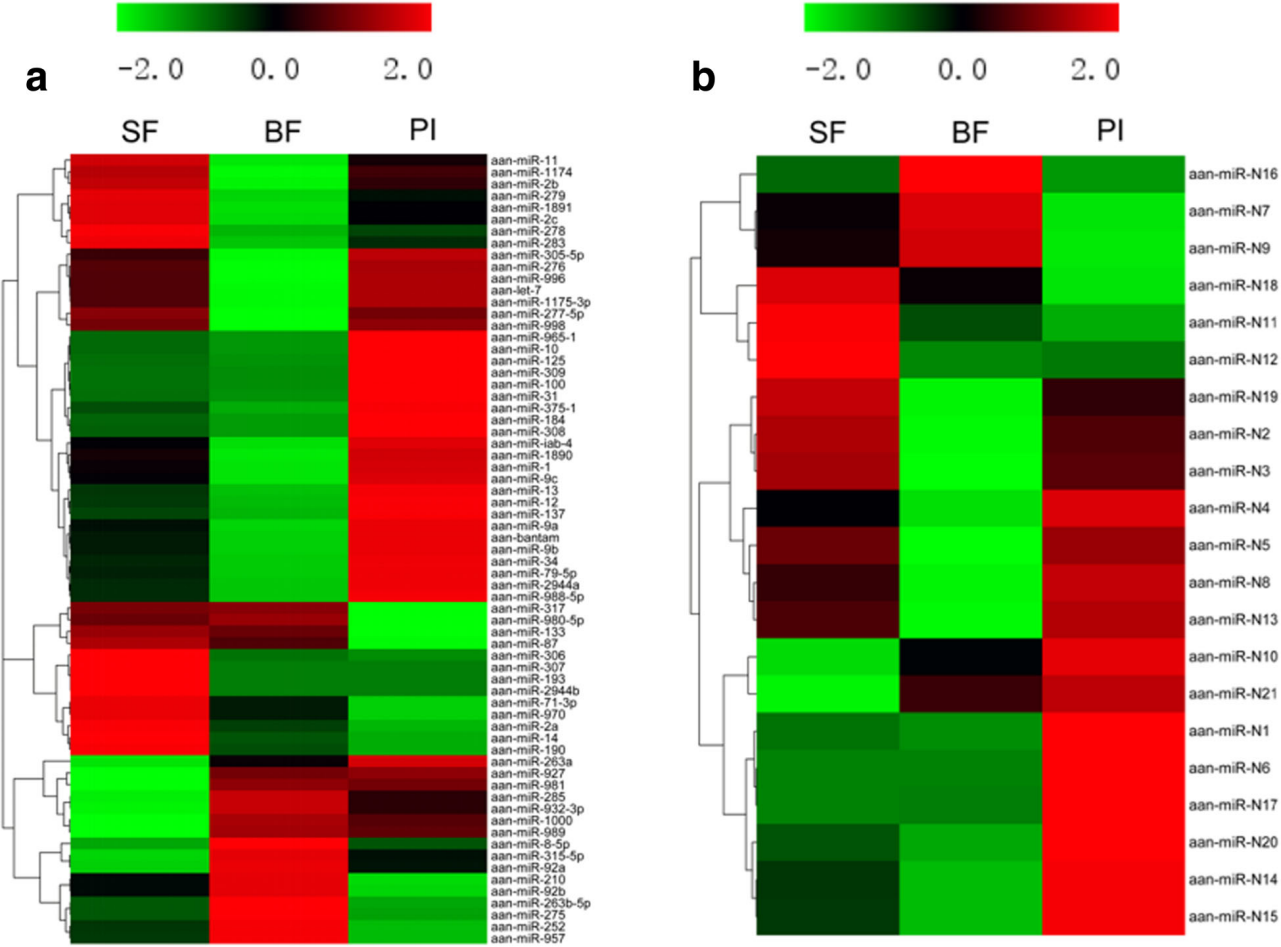
Heatmap of differential expressed miRNAs in the *An. anthropophagus* midgut. Expression profile of known miRNAs (**a**) and novel miRNAs (**b**) in sugar-feeding midgut (SF), blood-feeding midgut (BF) and *Plasmodium*-infected midguts (PI). Colour scale-bar from light green to *dark red* indicates relative increase in miRNA expression

To investigate the miRNAs regulated in blood-feeding and *Plasmodium* infection, the TPM value of individual miRNA between BF and SF, PI and BF were compared in the form of the fold change (Fig. 2). The set of significantly regulated miRNAs were selected on the basis of their *P*-value < 0.05, fold change ≥ 2 and TPM ≥10 (Additional file 1: Table S1 and Additional file 2: Table S2). With respect to BF library, six known miRNAs (miR-252, miR-315-5p, miR-92a, miR-92b, miR-932-3p and miR-989) and three novel miRNAs (miR-N10, miR-N16 and miR-N21) showed significantly upregulated expression compared with SF. Especially, the expression levels for miR-92a, miR-932-3p, miR-N16 and miR-N21 were enhanced more than 5-fold after blood-feeding. Meanwhile, the expression of 10 miRNAs (miR-1, miR-1175-3p, miR-12, miR-13, miR-276, miR-279, miR-283, miR-2b, miR-996 and miR-N3) was found to be significantly downregulated after blood-feeding (Tables 2 and 3).

In the case of the PI library, 9 known miRNAs (miR-1, miR100, miR-12, miR-13, miR-263a, miR-276, miR-308-5p, miR-31, miR-iab-4) and 4 novel miRNAs (miR-N1, miR-N6, miR-N14 and miR-N20) exhibited significantly upregulated expression. Notably, the TPM value of aan-miR-N16 was almost 150-fold enhanced in PI midgut compared with BF midgut. Meanwhile, the expression of miR-31 was enhanced more than 10-fold after *Plasmodium* infection. On the other hand, the expression levels of 9 known (miR-133, miR-14, miR-210, miR-252, miR-2a, miR-275, miR-92b, miR-957 and miR-980) and 2 novel miRNAs (miR-N16 and miR-N18) were significantly downregulated in the *Plasmodium* infection midgut. In particular, the TPM value for miR-252 was 191 in BF midgut but zero in the PI midgut (Tables 2 and 3).

Furthermore, several miRNA exhibited different expression patterns in BF and PI midguts. For example, four miRNAs including miR-1, miR-12, miR-13 and miR-276 were significantly downregulated in the BF midguts but upregulated in the PI midguts. Conversely, the expression of miR-252 and miR-N16 showed significant upregulation in the BF midguts but were downregulated in the PI midguts (Tables 2 and 3). These data suggest that the same miRNAs may play different roles in the blood-feeding and *Plasmodium* infection midguts.

### Confirmation of mosquito miRNAs

**Fig. 3:**
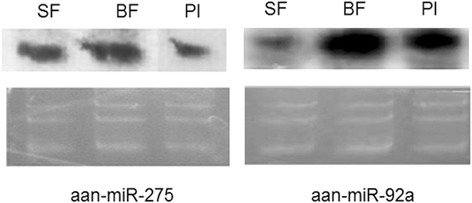
Northern blot of differentially expressed miRNAs in the *An. anthropophagus* midguts. *Abbreviations*: SF, sugar-feeding midgut; BF, blood-feeding midgut; PI, *Plasmodium*-infected midgut

Having identified the microRNAs expression patterns of *An. anthropophagus* midguts after blood-feeding and *Plasmodium* infection, Northern hybridization was performed to validate some of these miRNAs. The representative abundant miRNA (miR-275 and miR-92a) were selected in the Northern blot analysis. Total RNA from SF, BF and PI 48 h were probed using locked nucleic acid (LNA) probe. The expression patterns of miR-275 and miR-92a in SF, BF and PI are shown in Fig. 3. The northern signals at ~20 nt indicated that the expression level of miR-92a is upregulated to over 5-fold after blood-feeding, and miR-275 is downregulated to more than 3-fold after *Plasmodium* infection, which is consistent with the sequencing results.

### Target prediction and network analysis

**Fig. 4:**
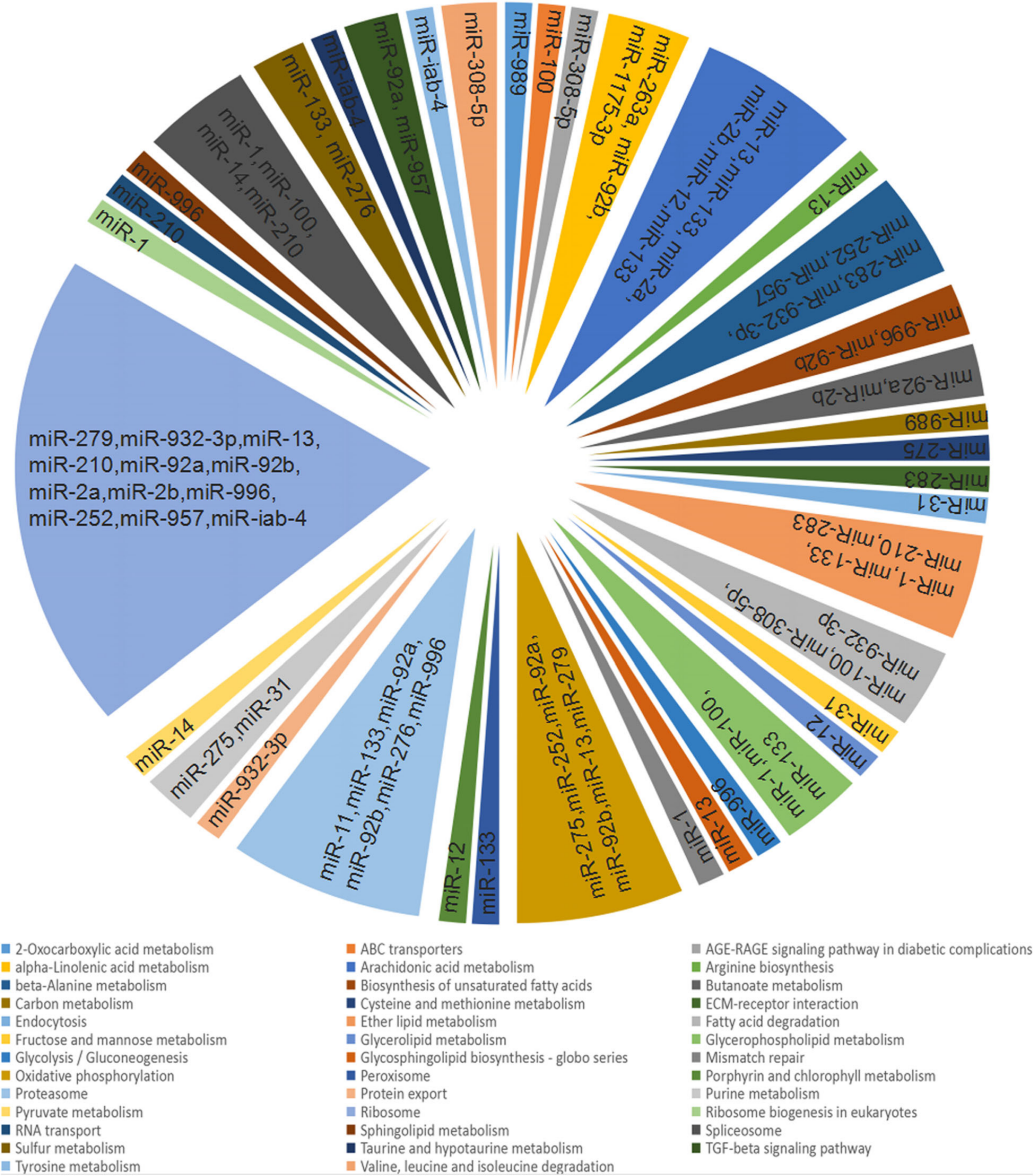
KOBAS analysis of miRNA targets. miRNA targets involved in blood-feeding and *Plasmodium* infection were predicted by RNA hybrid. Pie chart represents the pathway targeted by the miRNAs which are listed in the pie area

Target prediction were carried out to understand the putative function of regulated miRNAs by using RNA hybrid (*P*-value < 0.05). All the mRNA targets are derived from the orthologous genes of *An. gambiae* in the Vector Base. Maximum numbers of mRNA targets were predicted for miR-92a (*n* = 744). The targets were further analyzed by KOBAS, and a total of 38 different pathways were regulated by miRNAs after blood-feeding and *Plasmodium* infection (Fig. 4). In the upregulated miRNA after blood-feeding, miR-252 and miR-92a were identified to target oxidative phosphorylation and peroxisome pathway. miR-92a was also found to target glycolysis, proteasome, ribosome and TGF-beta signaling pathway. Meanwhile, ribosome was commonly targeted by the downregulated miRNAs including miR-13, miR-279 and miR-2b in BF midguts. After *Plasmodium* infection, endocytosis and fructose mannose metabolism pathway is specifically targeted by miR-31. Spliceosome was common between miR100 and miR-14. In the downregulated miRNA, miR-210 and miR-275 targeted RNA transport and purine metabolism separately (Fig. 4).

The interaction networks of miRNA:mRNA targets were generated. In BF midguts, AGAP000348-RA and AGAP000120-RA were targeted by downregulated miR-1. A total of 493 mRNA targets were regulated by upregulated miR-989, of which AGAP007839-RA, AGAP0004451-RA and AGAP008345-RA were targeted by two or more upregulated and downregulated miRNAs (Additional file 3: Figure S1). Furthermore, 573 mRNAs were targeted by upregulated miR-31 in the PI midguts. Three mRNA (AGAP000204-RA, AGAP009077-RA and AGAP010306-RA) were targeted by downregulated miR-957 (Additional file 4: Figure S2). Further investigations are important to understand the role of these mRNAs during blood-feeding and *Plasmodium* infection.

## Discussion

*Anopheles* mosquitoes are the main vector for the transmission of malaria, and the invasion of midguts is one of the most critical steps for the survival and development of *Plasmodium* [21]. Because of the lack of an adaptive immune system, RNA interference (RNAi) is the most important and primary defense employed by mosquitoes to protect themselves from pathogens [22, 23]. miRNAs are the most important part of RNA interference, and some of them have been proven to be involved in fighting against virus and parasite infections [21–24]. In a previous study, we have investigated the miRNA profiles of *An. anthropophagus*, and discovered that several miRNAs exhibit sexual differences and stage-specific functions [12]. However, their role in *Plasmodium* infection is poor understood. This study was conducted to identify and elucidate role of miRNAs after blood-feeding and *Plasmodium* infection in *An. anthropophagus* midguts.

In the present study, the expression profiles of miRNA at 48 h after blood-feeding and *Plasmodium* infection were investigated using small RNA sequencing; sugar-feeding midguts were taken as the control. Compared with sugar feeding, there is a significant downregulation for the percent of miRNA library (20–23 nt) after blood-feeding and *Plasmodium* infection. The similar downregulation expression profiling of miRNAs was also reported in the parasitized blood-feeding midguts of *Anopheles stephensi* [18] suggested that pathogens including *Plasmodium* and viruses invade mosquitoes midguts by inhibiting or downregulating the miRNA expression [22–24].

Blood-feeding is a critical physiological activity for the mosquito and its ability to transmit disease [25]. Female mosquitoes take blood meals to carry out egg production, and acquire pathogens such as malaria parasites and dengue viruses from an infected host [26–28]. Furthermore, mosquito biological characteristics are affected by triggering a cascade of gene regulatory events in the midgut after blood-feeding [28, 29]. In this study, the correlation between blood-feeding and miRNA expression profiles in *An. anthropophagus* midguts was investigated. We indentified nine significantly upregulated and ten downregulated expression miRNAs in the blood-feeding midguts compared with sugar feeding. For example, miR-92a exhibited a significant enhanced expression level after blood-feeding according to the sequencing and Northern blot results. Li et al. [11] reported that blood-feeding can induce the expression of miR-92a in the midgut of *Ae. aegypti* females. While *Wolbachia* infection can downregulate the expression of miR-92a in mosquito cell [30]. In our study, the expression of miR-92a is significantly upregulated in blood-feeding. By target prediction and networks analysis, miR92a was found to target 744 mRNAs and several pathways including the oxidative phosphorylation, proteasome, ribosome and TGF-beta singaling pathway. Our study may shed light on the possible roles of miR92a in *An. anthropophagus* mosquito physiology related to blood-feeding.

Midgut represents the first barrier for the pathogens to establish infection in mosquitoes [2, 4]. Pathogens such as parasites and endosymbiotic bacteria are known to alter host miRNA profiles for their invasion and development [15, 31]. For example, *Wolbachia* can induce the expression of miR-12 in *An. aegypti* mosquito cells to maintan the persistence of infection [31]. In our study, we indentified 13 upregulated miRNAs including miR-12. This finding suggests that miR-12 is also involved in *Plasmodium* infection, and the role of miR-12 in *Plasmodium* infection of *An. anthropophagus* need to be further elucidated. Meanwhile, we also found downregulated expression for 11 miRNA in *Plasmodium* infection midguts of *An. anthropophagus*, six of which including miR-14, miR-2a, miR-92b, miR-957, miR-980 and miR-275, were also shown to be downregulated in *Plasmodium*-infected *Anopheles stephensi* [18]*.* miR-275 is indispensable for blood digestion and egg development in the mosquito *Ae. aegypti* [32]. In this study, we confirmed that miR-275 is also involved in *Plasmodium* infection of *An. anthropophagus* by sequencing and further Northern blot analysis.

## Conclusions

In conclusion, our study provides the significant experimental data on the expression profile of microRNAs in *An. anthropophagus* midgut after blood-feeding and *Plasmodium* infection. Differentially expressed miRNA in SF, BF and PI were identified by small RNA sequencing, and further validated by Northern blot. By comparative analyzing differentially expression levels of the miRNA in sugar-feeding, blood-feeding and *Plasmodium* infection, we found several significant miRNAs involved in the interaction of mosquito host and parasite *Plasmodium*. Our study provides novel regulated miRNAs information of *An. anthropophagus* during blood-feeding and parasite infection. Elucidation of regulated miRNA functions will provide strong foundation for better understanding of the biology of the mosquitoes and mosquito-parasite interactions.

## Additional files

Additional file 1: Table S1. Known miRNAs in *Anopheles anthropophagus* midgut. (XLSX 14 kb)
Additional file 2: Table S2. Novel miRNAs in *Anopheles anthropophagus* midgut. (XLS 25 kb)
Additional file 3: Figure S1. Interaction network of regulated miRNA and mRNA targets after blood-feeding. (JPG 1651 kb)
Additional file 4: Figure S2. Interaction network of regulated miRNA and mRNA trargets after *Plasmodium* infection. (JPG 1638 kb)

## Abbreviations

BF: Blood-feeding
FPKM: Fragments Per Kilobase of transcript per Million mapped reads
miRNAs: microRNAs
PI: *Plasmodium* infection
RNAi: RNA interference
SF: Sugar feeding
