# Supplementary figures and images for: Comparative expression profile of microRNAs in Anopheles anthropophagus midgut after blood-feeding and Plasmodium infection
Source: Parasit Vectors. 2017 Feb 16;10:86. doi: 10.1186/s13071-017-2027-6 (PMC5314681; doi:10.1186/s13071-017-2027-6)

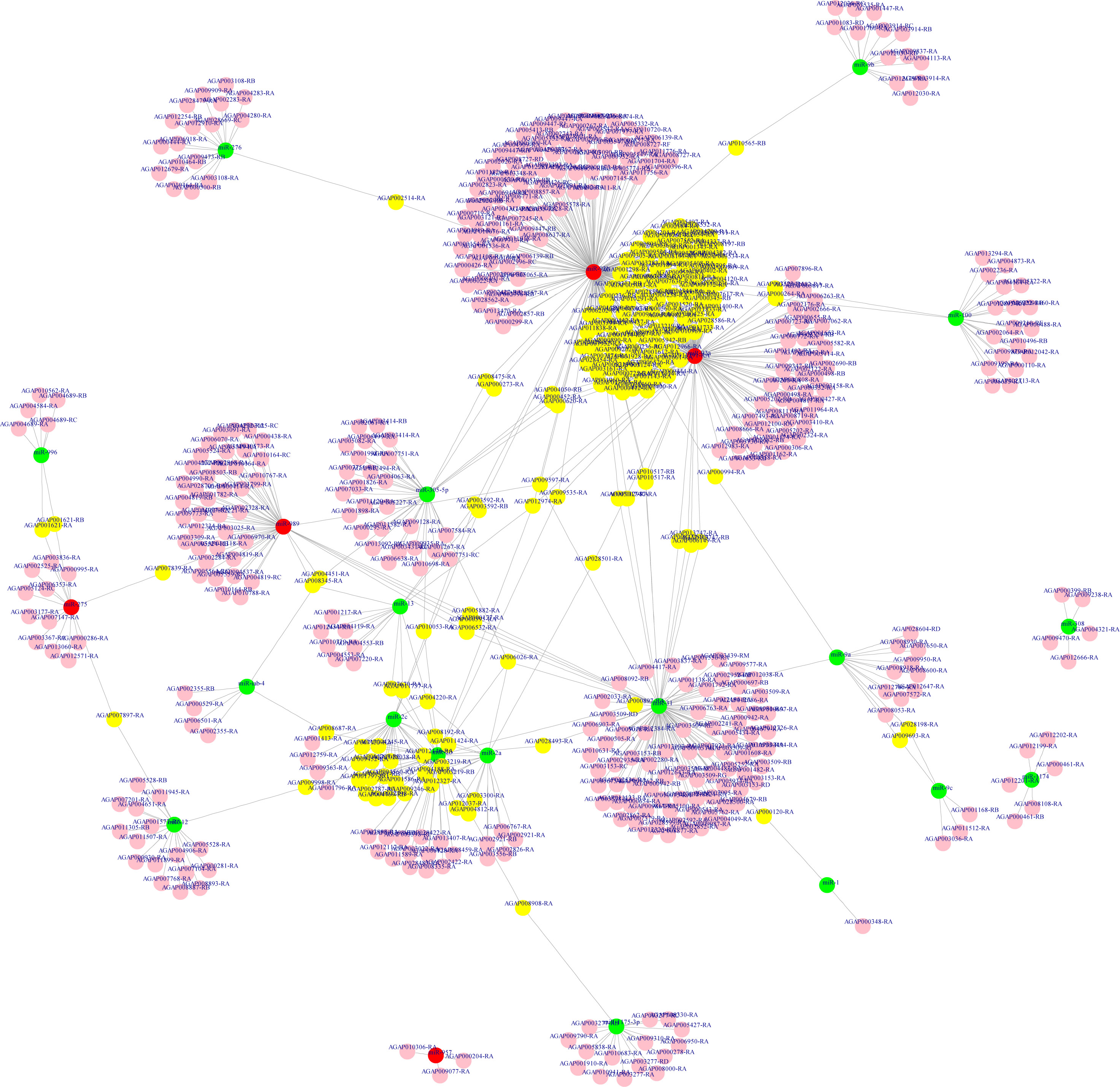

Supplement: Additional file 3: Figure S1. — Interaction network of regulated miRNA and mRNA targets after blood-feeding. (JPG 1651 kb) [file 13071_2017_2027_MOESM3_ESM.jpg]

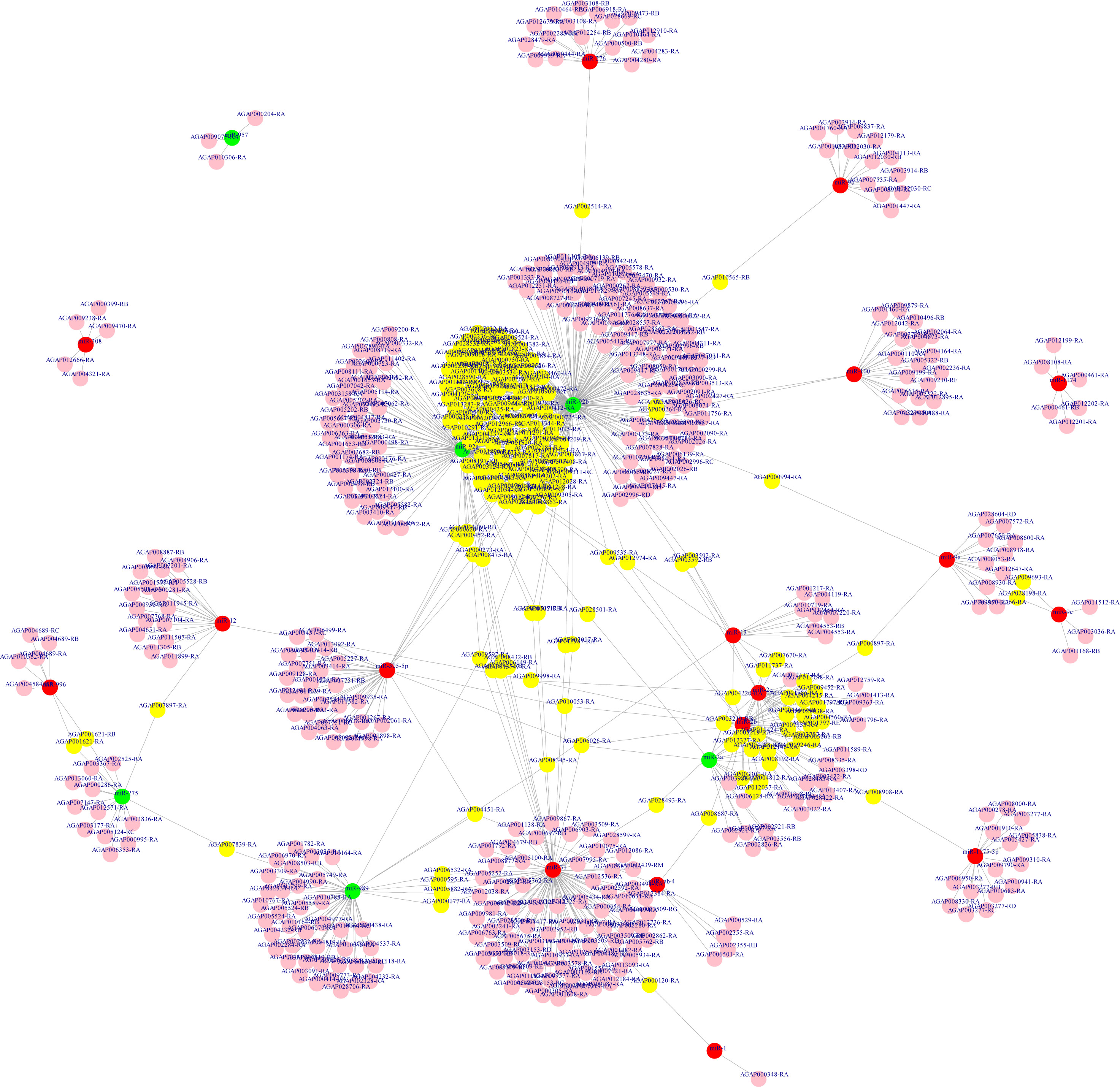

Supplement: Additional file 4: Figure S2. — Interaction network of regulated miRNA and mRNA trargets after Plasmodium infection. (JPG 1638 kb) [file 13071_2017_2027_MOESM4_ESM.jpg]
